# High immunoglobulin-M levels to oxidation-specific epitopes are associated with lower risk of acute myocardial infarction

**DOI:** 10.1016/j.jlr.2023.100391

**Published:** 2023-05-19

**Authors:** Adam Taleb, Peter Willeit, Shahzada Amir, Thomas Perkmann, Maria Ozsvar Kozma, Martin L. Watzenböck, Christoph J. Binder, Joseph L. Witztum, Sotirios Tsimikas

**Affiliations:** 1 Division of Cardiovascular Medicine, Vascular Medicine Program, University of California San Diego, La Jolla, CA, USA; 2 Department of Neurology, Medical University of Innsbruck, Innsbruck, Austria; 3 Department of Public Health and Primary Care, University of Cambridge, Cambridge, United Kingdom; 4 Division of Nuclear Medicine, Department of Biomedical Imaging and Image-Guided Therapy, Medical University of Vienna, Vienna, Austria; 5 Department of Laboratory Medicine, Medical University of Vienna, Vienna Austria; 6 Division of Endocrinology and Metabolism, Department of Medicine, University of California San Diego, La Jolla, CA, USA

**Keywords:** oxidation, immunoglobulin, myocardial infarction

## Abstract

Immunoglobulin M (IgM) autoantibodies to oxidation-specific epitopes (OSEs) can be present at birth and protect against atherosclerosis in experimental models. This study sought to determine whether high titers of IgM titers to OSE (IgM OSE) are associated with a lower risk of acute myocardial infarction (AMI) in humans. IgM to malondialdehyde (MDA)-LDL, phosphocholine-modified BSA, IgM apolipoprotein B100-immune complexes, and a peptide mimotope of MDA were measured within 24 h of first AMI in 4,559 patients and 4,617 age- and sex-matched controls in the Pakistan Risk of Myocardial Infarction Study. Multivariate-adjusted logistic regression was used to estimate odds ratio (OR) and 95% confidence interval for AMI. All four IgM OSEs were lower in AMI versus controls (*P* < 0.001 for all). Males, smokers and individuals with hypertension and diabetes had lower levels of all four IgM OSE than unaffected individuals (*P* < 0.001 for all). Compared to the lowest quintile, the highest quintiles of IgM MDA-LDL, phosphocholine-modified BSA, IgM apolipoprotein B100-immune complexes, and MDA mimotope P1 had a lower OR of AMI: OR (95% confidence interval) of 0.67 (0.58–0.77), 0.64 (0.56–0.73), 0.70 (0.61–0.80) and 0.72 (0.62–0.82) (*P* < 0.001 for all), respectively. Upon the addition of IgM OSE to conventional risk factors, the C-statistic improved by 0.0062 (0.0028–0.0095) and net reclassification by 15.5% (11.4–19.6). These findings demonstrate that IgM OSE provides clinically meaningful information and supports the hypothesis that higher levels of IgM OSE may be protective against AMI.

Oxidation-specific epitopes (OSE) are generated during lipid peroxidation of unsaturated fatty acids present on lipoproteins, membranes of dying cells, and extracellular vesicles (1, 2). OSE represent danger-associated molecular patterns (DAMPs) (3) that create inflammation by upregulating the expression of pro-inflammatory genes in a variety of cell types, including endothelial cells, monocytes, and macrophages and inducing the release of cytokines such as IL1-β, TNFα and IL-6 (4, 5, 6, 7, 8, 9, 10). In some cases, OSE share molecular identity or mimicry with epitopes on pathogens and are denoted as pathogen-associated molecular patterns (PAMPs). Both adaptive and innate immune responses have been evolutionarily optimized to bind to and inactivate OSE. This is likely mediated through selective pressure of such OSE on PAMPs of infectious pathogens and DAMPs on apoptotic cells, OxLDL, and other lipid peroxidation products. Pattern recognition receptors (PRRs), such as innate IgM natural antibodies (NAbs), C-reactive protein, and scavenger receptors on macrophages, may recognize OSE.

A consequence of lipid peroxidation is the generation of subtle conformational changes that occur on lipoproteins, cells, and microvesicles, such as the formation of malondialdehyde (MDA) adducts on lysine residues of proteins (11). Phosphocholine (PC)-containing oxidized phospholipids (PC-OxPL) may be generated when the polyunsaturated fatty acid side chain of an unoxidized phospholipid is oxidized, resulting in a change in conformation that generates a DAMP neoepitope (12). IgM autoantibodies specific for OSE are thought to reflect innate NAbs that develop during fetal development and are present at birth or shortly thereafter even in the absence of antigen exposure, and their antibody variable genes have high homology to germline sequences with few if any non-templated nucleotide additions (13). Detection of elevated levels of OSE in the circulation, particularly PC-containing OxPL, is associated with increased cardiovascular risk (14, 15). In parallel, extracellular vesicles carrying OSE are enriched at the culprit lesions of coronary arteries in patients presenting with acute coronary syndrome and represent important targets of OSE-IgM (16). The presence of IgM autoantibodies in plasma, a subset of which reflect innate NAbs, as well as circulating immune complexes containing OSE-antibodies can reflect various manifestations of CVD (17, 18, 19, 20, 21).

The role of IgM OSE levels in patients with acute MI (AMI) is not well defined. We hypothesized that high titers of IgM autoantibodies binding to a variety of OSE would be associated with reduced risk of AMI in the PROMIS study. In addition, we tested the ability of a novel antigen representing an MDA-mimotope as one of the 4 IgM OSE, to reflect a higher risk of cardiovascular events.

## Materials and methods

### PROMIS study cohort

The PROMIS Study is a case–control study of patients with confirmed first-onset AMI and included patients aged 30–80 years who were recruited from seven centers in five cities in Pakistan (22, 23). Patients were eligible if they had an AMI and had characteristic symptoms of an event within 24 h of hospital admission, typical changes on electrocardiogram (ECG), and a positive troponin-I test. Controls were hospital visitors, concurrently identified in the same hospitals as patients with myocardial infarction, who did not have a self-reported history of cardiovascular disease or ECG changes consistent with a previous myocardial infarction. Controls were matched to myocardial infarction cases by sex and age (5-year bands). Non-fasting blood samples (with the time since the last meal and time since onset of chest symptoms recorded) were taken from each participant and centrifuged within 45 min of venipuncture, and the plasma was immediately stored at −80°C. Exclusion criteria included: *1*) prior history of cardiovascular disease (including self-reported MI, angina, coronary revascularization, stroke, transient ischemic attack, or peripheral vascular disease, and, in some cases, presentation with cardiogenic shock); *2*) a history of a viral or bacterial infection in the previous 2 weeks; *3*) documented chronic conditions, such as malignancy, any chronic infection, leprosy, malaria or other bacterial/parasitic infections, chronic inflammatory disorders, hepatitis or renal failure on past medical history; *4*) pregnancy; or *5*) refusal to give consent. The PROMIS has received approval from the relevant research ethics committee of each of the institutions involved in participant recruitment (22). The human studies reported in the article abide by the Declaration of Helsinki principles.

### IgM OSE assays

The levels of IgM autoantibodies binding to malondialdehyde-low density lipoprotein (IgM MDA-LDL) and the IgM apolipoprotein B100-immune complexes (IgM apoB-IC) were detected in human plasma with the use of a chemiluminescent ELISA as previously described (20).

In brief, to detect IgM to MDA-LDL, MDA-LDL (5 μg/ml) was coated on microtiter well plates, plasma (1:200 dilution) was added, and IgG or IgG autoantibodies binding to MDA-LDL was detected with alkaline phosphatase–labeled goat anti-human IgG or IgM (Sigma). To detect ApoB-100 ICs, murine monoclonal antibody MB47 recognizing human apoB-100 was plated to bind a saturating amount of human apoB-100. Plasma (1:50 dilution) was added and IgM autoantibodies binding to the captured apoB-100 (i.e., apoB-IC) were detected with alkaline phosphatase labeled goat anti-human IgM. To detect IgM autoantibodies binding to phosphocholine-bovine serum albumin (IgM PC-BSA), phosphocholine-modified bovine serum albumin (PC-BSA, 50 μl PC-BSA at 1 μg/ml) (Biosearch Technologies) was plated overnight in 96-well microtiter plates at 4°C. After washing, human plasma samples diluted at 1:200 with 1% BSA/TBS were added. A goat anti-human IgM antibody conjugated with alkaline phosphatase (Sigma-Aldrich) was used to detect IgM bound to the PC-BSA, Lumi-Phos (Lumigen) added, and the plates were read in a chemiluminescent detector.

IgM titers against MDA mimotope P1 were measured as previously described (24). The P1 peptide is a dodecamer linear peptide with sequence HSWTNSWMATFL. The P1 MDA peptide mimotope (10 μg/ml) was coated on microtiter 386-well plates, plasma (1:1,600 dilution) was added, and IgM autoantibodies binding to P1 was detected with alkaline phosphatase labeled goat anti-human IgM (Sigma). In prior studies (24, 25, 26, 27, 28), P1 bound specifically to murine and human anti-MDA monoclonal antibodies, and was found to mimic MDA epitopes on the surface of apoptotic cells. The presence of IgM autoantibodies to P1 mimotope was noted in sera of healthy controls and patients with MI and stable angina pectoris undergoing percutaneous coronary intervention, and the titers of P1 autoantibodies correlated significantly with respective antibody titers against MDA-LDL.

High and low standards for each variable were used on each plate to detect potential variations among plates, and the results were reported in relative light units (RLUs) in 100 ms. The intra-assay coefficients of variation for all the assays were 6%–12%.

### Total IgM, IgG, and IgA measurements in EPIC-Norfolk as control variables

To provide evidence that changes in IgM OSE were specific to the risk of MI and do not reflect generalized non-specific increases, total IgM levels were measured as a control variable. EPIC-Norfolk is a prospective nested case–control study among participants of the cohort who did not report a history of heart attack or stroke at the baseline clinic visit. Cases were people who developed CAD during follow-up through 2003 as previously described (29). Control participants were apparently healthy study participants who remained free of any cardiovascular disease during 7.4 years of follow-up. Two controls were matched to each case by sex, age (within 5 years), and date of visit (within 3 months). A total of 2,511 blood samples were available, including 1,747 controls and 764 cases. Levels of total IgM, IgG, and IgA were performed with nephelometry using the BN II System (Siemens). IgG and IgM MDA-LDL and ApoB-IC were previously reported in EPIC-Norfolk (20). The EPIC-Norfolk study received approval from the relevant research ethics committee of each of the institutions involved in participant recruitment (30).

### Statistical analysis

Continuous variables were presented as means ± standard deviations (SD) or medians and interquartile range (IQR) and dichotomous variables as percentages. Differences in baseline attributes between participants were analyzed with Χ^2^-test for categorical variables and Mann Whitney U test was used for continuous variables. Correlations between variables were determined with the Spearman test. Variables with a skewed distribution were log-transformed before being used as continuous variables in analyses. Multivariate-adjusted binary logistic regression models were used to estimate the associations between IgM OSE and risk of AMI as ratios (OR) and corresponding 95% confidence intervals (CI), by quintiles and per standard deviation, with adjustment for sex, age, smoking status, the presence or absence of hypertension and diabetes, LDL-C, HDL-C, and log_2_ triglycerides.

The predictive values of IgM OSE were quantified by: (i) comparing c-statistics of all risk factors in the multivariable analysis, and then adding individual IgM OSE alone and then all together and (ii) calculating the continuous net reclassification improvement (which is suitable for case-control studies because it does not require knowledge about the underlying disease incidence) (31).

Formal tests of interaction were used to investigate whether associations of IgM biomarkers with AMI differed according to other participant characteristics. Statistical analyses were performed using IBM SPSS software (version 24). *P* value < 0.05 was considered significant.

## Results

### Demographic variables of cases with AMI and controls

Demographic variables are shown in Table 1. Compared to controls, cases wit AMI had a higher incidence of hypertension (32.8% vs. 30.6%, *P* < 0.001), diabetes (19.6% vs. 13.4%, *P* < 0.001), were more likely to smoke (49.6% vs. 31.6%, *P* < 0.001), and had higher total cholesterol levels (191.7 mg/dl vs. 183.6 mg/dl, *P* < 0.001) and LDL-C levels (122.9 mg/dl vs. 109.3 mg/dl, *P* < 0.001).

**Table 1:** Baseline demographic and biochemical characteristics of the study groups

Characteristics	Controls N = 4,617	AMI N = 4,559	*P*
Males	3,710 (80.4%)	3,785 (83.0%)	Matched
Age (years)	53.8 (9.3)	53.9 (10.4)	Matched
Hypertension	1,412 (30.6%)	1,495 (32.8%)	<0.001
Diabetes Mellitus	618 (13.4%)	895 (19.6%)	<0.001
Tobacco Use, Current	1,461 (31.6%)	2,137 (46.9%)	<0.001
BMI (kg/m^2^)	26.2 (5.4)	25.1 (3.9)	<0.001
Total Cholesterol (mg/dl)	183.6 (49.8)	191.7 (50.7)	<0.001
LDL-C (mg/dl)	109.3 (39.7)	122.9 (43.7)	<0.001
Triglycerides (mg/dl)	180 (126–263)	163 (112–237)	<0.001
HDL-C (mg/dl)	35.2 (10.7)	34.9 (10.8)	0.25
IgM MDA-LDL (RLU)	15,541 (11,048–21,427)	14,271 (10,141–19,775)	<0.001
IgM PC-BSA (RLU)	12,881 (7,971–19,770)	11,207 (7,041–17,608)	<0.001
IgM ApoB-IC (RLU)	1,768 (1,199–2,630)	1,607 (1,096–2,417)	<0.001
IgM MDA Mimotope P1 (RLU)	4,362 (2,547–7,908)	3,840 (2,211–7,039)	<0.001

kg/m^2^, kilogram per square meter; mg/dl, milligram per deciliter; RLU, relative light units.

Age, BMI, total cholesterol, triglycerides, LDL-C, HDL-C are reported as mean (SD). Tobacco history, DM, HTN: number of individuals are reported as a percentage. IgM MDA-LDL, IgM apoB-IC, IgM PC-BSA and IgM Mimotope P1 are reported as median (IQR).

### Baseline IgM OSE values in cases with AMI versus controls

Relative to controls, cases with AMI had significantly lower median (IQR) levels of IgM MDA-LDL autoantibodies [14,271 (10,141–19,775) versus 15,541 (11,048–21,427) RLU, *P* < 0.001], IgM PC-BSA autoantibodies [11,207 (7,041–17,608) versus 12,881 (7,971–19,770) RLU, *P* < 0.001], IgM Apo-B-IC [1,607 (1,096–2,417) versus 1,779 (1,096–2,417) RLU, *P* < 0.001], and IgM MDA Mimotope P1 [3,840 (2,211–7,039) versus 4,362 (2,547–7,908) RLU, *P* < 0.001 (Table 1).

### Risk of AMI according to IgM OSE levels

A significant and inverse association was present for all 4 oxidative IgM OSE and AMI following multivariable adjustment (Table 2). Using quintile 1 (Q1) as the reference group, progressively declining OR were present for all oxidative IgM OSE, with Q5 OR (95% CI) 0.67 (0.58–77) for IgM MDA-LDL, 0.64 (0.56–0.73) for IgM PC-BSA, 0.70 (0.61–0.80) for IgM apoB-IC and 0.72 (0.62–0.82) for IgM MDA Mimotope P1 (*P* < 0.001 for all).

**Table 2:** Risk of AMI according to quintiles of IgM MDA-LDL, IgM PC-BSA, IgM apoB-IC and IgM MDA mimotope P1

	IgM MDA-LDL	IgM PC-BSA	IgM apoB-IC	IgM MDA Mimotope P1
Across Categories				
OR (95% CI) versus reference				
Quintile 1	1.00 [Ref]	1.00 [Ref]	1.00 [Ref]	1.00 [Ref]
Quintile 2	0.90 (0.79–1.03)	0.89 (0.77–1.02)	0.90 (0.79–1.03)	0.82 (0.72–0.94)
Quintile 3	0.80 (0.70–0.92)	0.86 (0.75–0.98)	0.80 (0.70–0.91)	0.76 (0.67–0.87)
Quintile 4	0.75 (0.65–0.86)	0.75 (0.66–0.86)	0.75 (0.65–0.86)	0.73 (0.64–0.84)
Quintile 5	0.67 (0.58–0.77)	0.64 (0.56–0.73)	0.70 (0.61–0.80)	0.72 (0.62–0.82)
*P* value for trend	<0.001	<0.001	<0.001	<0.001
Per SD higher value				
OR (95% CI)	0.90 (0.87–0.94)	0.83 (0.79–0.87)	0.90 (0.85–0.95)	0.94 (0.90–0.98)
*P* value	<0.001	<0.001	<0.001	<0.001

Odds ratios are adjusted for age, gender, HTN, smoking, DM, log_2_ triglycerides, HDL-C, and LDL-C.

To rule out a potential acute-phase response in IgM OSE levels that may have influenced findings, the distribution of IgM OSE levels was plotted at each hour over 24 h since the onset of chest pain in the cases. The IgM OSE levels were flat over 24 h (supplemental Fig. S1).

The shape of the association of IgM OSE to myocardial infarction was analyzed in more detail by deciles. The data reveals that the odds ratios for myocardial infarction decrease continuously over most deciles, including all deciles in IgM PC-BSA (supplemental Fig. S2). There does not appear to be evidence for a threshold at a specific level.

To rule out a non-specific effect of total IgM titers driving this association, an additional control group was used from the EPIC-Norfolk case–control study. Total IgM titers were measured and OR for CAD determine with the hypothesis that the association would be null. In age, sex-, and smoking-adjusted analyses, there was no significant association of total IgM, IgG, and IgA and the presence of CAD. Details of these analyses are shown in the Supplementary Appendix Table.

### Interaction tests of the associations of IgM OSE according to clinical characteristics

**Fig. 1:**
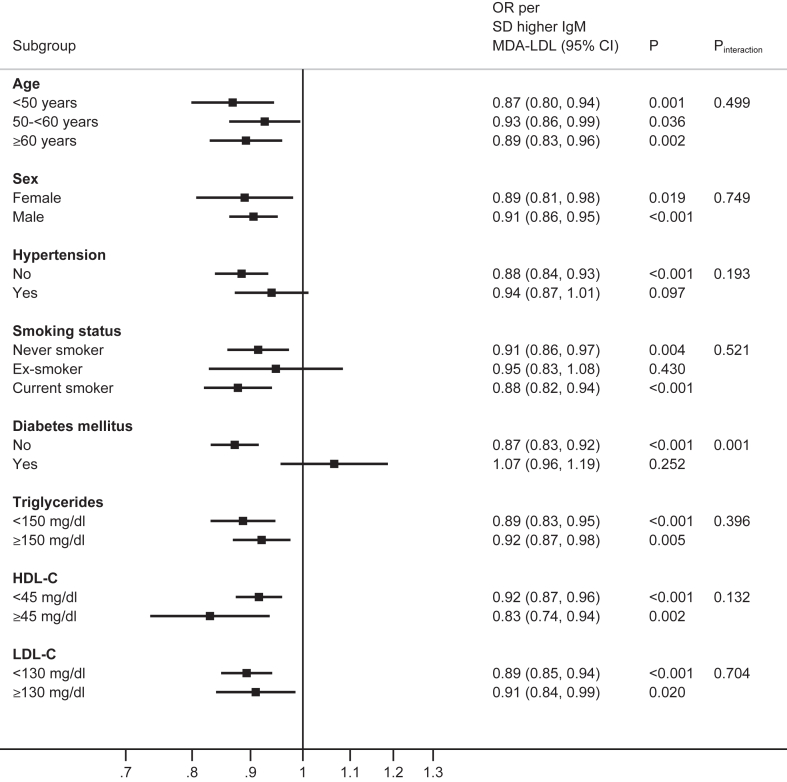
Interaction test analyses of subgroups of study participants in relationship with AMI by IgM MDA-LDL.

**Fig. 2:**
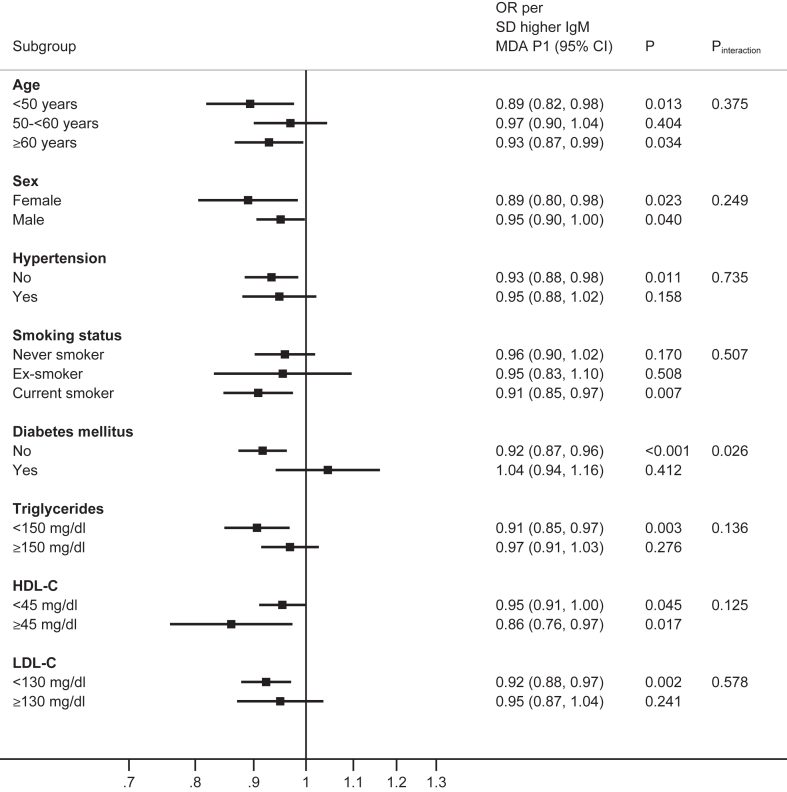
Interaction test analyses of subgroups of study participants in relationship with AMI by IgM MDA P1 mimotope

**Fig. 3:**
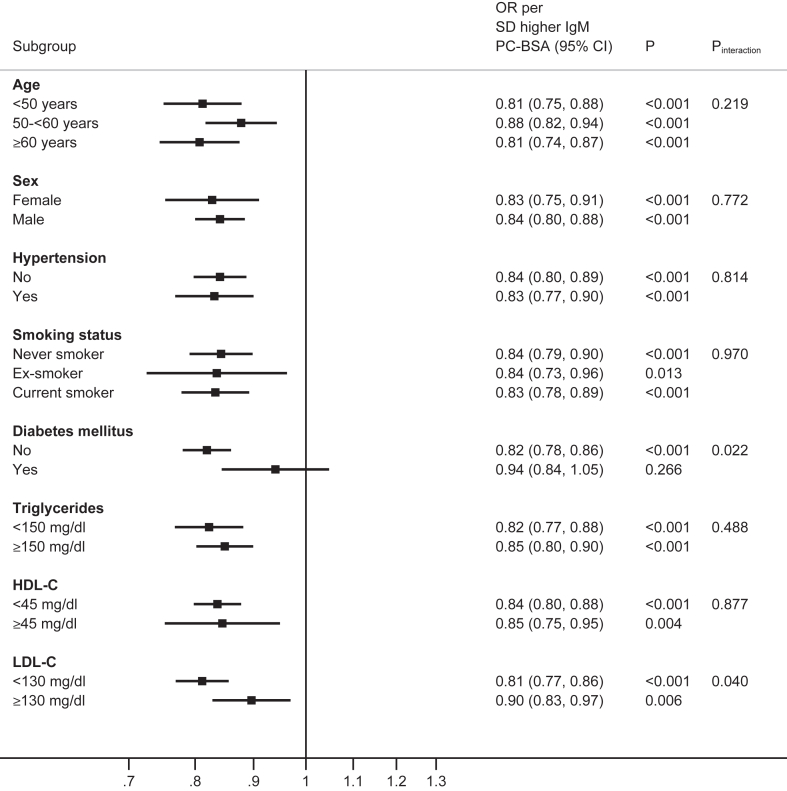
Interaction test analyses of subgroups of study participants in relationship with AMI by IgM PC-BSA.

**Fig. 4:**
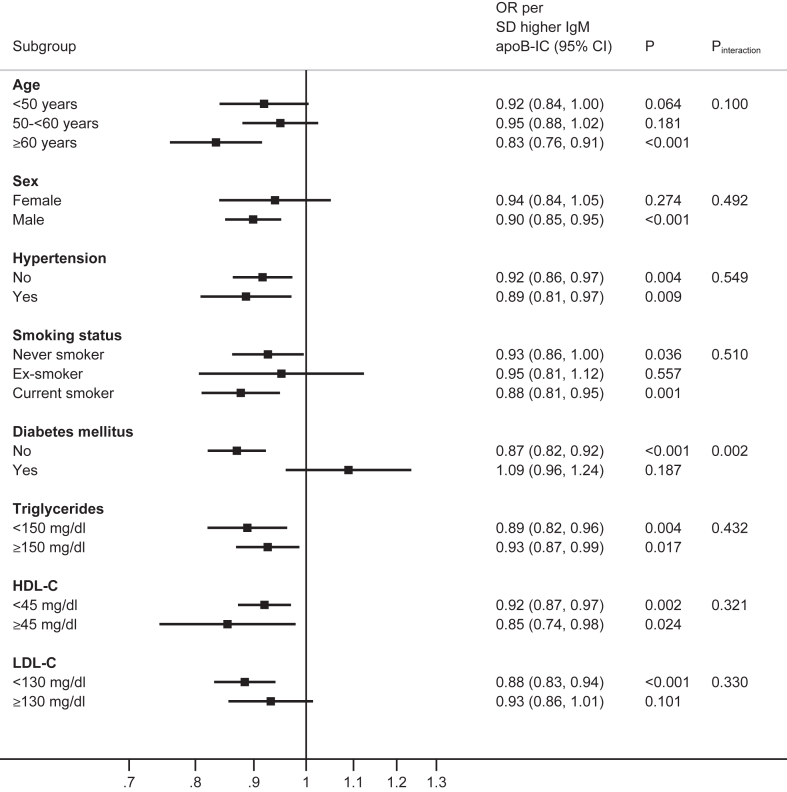
Interaction test analyses of subgroups of study subjects in relationship with AMI by IgM apoB-IC.

The effect modification association of IgM OSE with AMI status and participant characteristics were analyzed for age, sex, hypertension, smoking status, diabetes, triglycerides, HDL-C, and LDL-C. Analyzed as OR (95% CI) per 1 SD for each of four IgM OSE, there were relatively consistent effects across age, sex, hypertension, and smoking status, and by triglyceride <150 versus ≥ 150 mg/dl, HDL-C <45 versus ≥ 45 mg/dl and LDL-C <130 versus ≥ 130 mg/dl thresholds, without evidence of effect modification (*P* interaction >0.05). However, there was evidence of effect modification by diabetes status for all four IgM OSE, with significant interaction tests (IgM MDA-LDL *P* interaction = 0.001, IgM MDA-P1 mimotope *P* interaction =0.026, IgM PC-BSA *P* interaction = 0.022, IgM apoB-IC *P* interaction = 0.002 (Fig. 1, Fig. 2, Fig. 3, Fig. 4).

### Predictive models for AMI with IgM OSE

C-statistics are shown in Table 3. Risk factors alone (age, gender, BMI, HTN, smoking and DM status, triglycerides, HDL-C, and LDL-C) gave a c-statistic of 0.667. The addition of each oxidative IgM OSE yielded significant improvements in the C-statistic (Table 3); the addition of all four IgM OSE s increased the C-statistic to 0.674, corresponding to an improvement by 0.0062 (0.0028–0.0095). The continuous net reclassification improvement was +15.5% (11.4, 19.6) when adding all four IgM OSE.

**Table 3:** Predictive value of AMI according to levels of IgM OSE added to conventional risk factors

	C-statistic (95% CI)	C-statistic Change (95% CI)	Continuous net Reclassification Index (95% CI)
Model with conventional risk factors	0.667 (0.656, 0.678)	[Ref]	[Ref]
+ IgM MDA-LDL	0.671 (0.660, 0.682)	0.0034 (0.0007, 0.0061)∗	+12.5% (8.4, 16.6)∗∗∗
+ IgM PC-BSA	0.672 (0.661, 0.683)	0.0045 (0.0016, 0.0075)∗∗	+14.8% (10.7, 18.9)∗∗∗
+ IgM apoB-IC	0.670 (0.660, 0.681)	0.0031 (0.0006, 0.0056)∗	+11.5% (7.4, 15.6)∗∗∗
+ IgM MDA Mimotope P1	0.671 (0.660, 0.682)	0.0031 (0.0008, 0.0054)∗∗	+10.8% (6.7, 14.9)∗∗∗
+ all four biomarkers	0.674 (0.663, 0.685)	0.0062 (0.0028, 0.0095)∗∗∗	+15.5% (11.4, 19.6)∗∗∗

All IgM OSE were entered as quintiles. Risk factors include age, gender, BMI, HTN, smoking, DM, triglycerides. HDL-C and LDL-C. ∗*P* ≤ 0.05. ∗∗*P* ≤ 0.01. ∗∗∗*P* ≤ 0.001.

### Correlations among IgM OSE and lipid variables

Table 4 shows the Spearman correlation among IgM OSE and the lipid panel in the entire group and in the control and AMI groups. In the entire group, all 4 IgM OSE had modest correlations with each other, but weak to absent correlations with individual variables of the lipid panel. Similar relationships were present in the control and AMI groups when analyzed separately.

**Table 4:** Spearman’s rho correlations and *P* values among IgM OSE and laboratory variables

	IgM PC-BSA	IgM MDA P1	IgM ApoB-IC	Total Cholesterol	LDL-C	HDL-C	TG
Entire Group							
IgM MDA-LDL	0.60	0.73	0.68	−0.03	−0.03	0.03	−0.05
	*P* < 0.001	*P* < 0.001	*P* < 0.001	*P* = 0.007	*P* = 0.003	*P* = 0.003	*P* < 0.001
IgM PC-BSA		0.51	0.61	−0.02	−0.03	0.02	−0.01
		*P* < 0.001	P0.001	*P* = 0.104	*P* = 0.004	*P* = 0.044	*P* = 0.461
IgM MDA P1			0.59	−0.05	−0.05	0.00	−0.04
			*P* < 0.001	*P* < 0.001	*P* < 0.001	*P* = 0.76	*P* < 0.001
IgM ApoB-IC				−0.04	−0.05	−0.00	−0.04
				*P* < 0.001	*P* < 0.001	*P*=0.76	*P* < 0.001
Control Group							
IgM MDA-LDL	0.62	0.74	0.69	−0.04	−0.03	0.04	−0.08
	*P* = 0.001	*P* = 0.001	*P* = 0.001	*P* = 0.015	*P* = 0.08	*P* = 0.015	*P* < 0.001
IgM PC-BSA		0.54	0.62	−0.04	−0.05	0.01	−0.03
		*P* = 0.001	*P* = 0.001	*P* = 0.005	*P* = 0.002	*P* = 0.70	*P* = 0.049
IgM MDA P1			0.62	−0.07	−0.06	0.13	−0.08
			*P* = 0.001	*P* < 0.001	*P* < 0.001	*P* = 0.38	*P* < 0.001
IgM ApoB-IC				−0.07	−0.07	−0.01	−0.05
				*P* < 0.001	*P* < 0.001	*P* = 0.38	*P* < 0.001
AMI Group							
IgM MDA-LDL	0.57	0.72	0.66	−0.01	−0.01	0.03	−0.03
	*P* = 0.001	*P* = 0.001	*P* = 0.001	*P* = 0.60	*P* = 0.50	*P* = 0.07	*P* = 0.05
IgM PC-BSA		0.48	0.60	0.02	0.02	0.04	−0.004
		*P* < 0.001	*P* = 0.001	*P* = 0.12	*P* = 0.29	*P* = 0.014	*P* = 0.77
IgM MDA P1			0.56	−0.01	−0.02	−0.01	−0.02
			*P* = 0.001	*P* = 0.46	*P* = 0.24	*P* = 0.65	*P* = 0.21
IgM ApoB-IC				−0.01	−0.02	−0.02	−0.04
				*P* = 0.40	*P* = 0.18	*P* = 0.22	*P* = 0.004

## Discussion

This study demonstrates that individuals with increased titers of 4 IgM OSE are associated with a linear and graded lower risk of AMI independent of traditional risk factors. In addition, these findings suggest that they may provide clinical utility by demonstrating improvements in risk discrimination (0.0062 increase in the C-statistic) and risk classification (15.5% increase in continuous net reclassification index). This study represents the strongest powered study with 4,559 cases of AMI and showed consistent effects in all 4 IgM OSE that were measured. These IgM OSE measured reflect both MDA and OxPL OSE, which are the most well-characterized epidemiologically for association with CVD events (14, 15, 17, 18, 19, 20, 21, 32), as well as experimentally in preclinical models for therapeutic effects (6, 10, 33, 34, 35, 36).

In this study, the association of IgM OSE was consistent among subgroups studied except patients with type 2 diabetes mellitus. Interestingly, there was evidence of effect modification in subjects with diabetes, where the inverse association was strongly maintained among patients without diabetes but not with diabetes. This suggests that any protection provided by IgM OSE is lost in subjects with diabetes, perhaps due to a heightened state of oxidative stress in diabetes (37) where the levels of IgG OSE are not sufficient for protection. It has also been previously shown in the Dallas Heart Study that IgM OSE levels progressively decline each decade from age 20 onward (38). This observation suggests that IgM OSE may not be sufficient to protect against MI in scenarios of heightened oxidative stress. In translational applications, to overcome excessive oxidative stress such as in diabetes mellitus or other clinical scenarios where natural IgM OSE are insufficient, exogenous administration of OSE-directed antibodies or overexpression by gene therapy approaches may be considered as has been shown in animal models (6, 10, 33, 34, 35, 36, 39, 40, 41, 42, 43, 44, 45).

Several smaller studies measuring levels in AMI or acute coronary syndromes (ACS) have shown similar findings with either AMI (18, 46, 47) or prognosis following ACS (48) with a variety of IgM OSE markers, including oxidized cardiolipin, phosphocholine, and apoB-immune complexes. In support of the predictive value of plasma measurements, it has been shown that OSE such as OxPL and MDA can be strongly present within plaque debris captured by distal protection devices during coronary, carotid, renal, and peripheral interventions (49) or in ruptured and thrombotic plaques from sudden death victims (50). In patients without prior cardiovascular disease followed long-term, higher titers of IgM OSE directed to a variety of epitopes, including advanced glycation end-products, phosphocholine, oxidized cardiolipin, MDA, and copper-oxidized LDL have been associated with a lower risk of cardiovascular disease in many (51, 52, 53, 54, 55, 56), but not in all studies (20).

We further demonstrate that the peptide mimotope of MDA, termed the P1 mimotope that has a defined amino acid structure and behaves chemically and immunologically like MDA or the more advanced but related epitope MDA-acetaldehyde (MAA) (24), provides relatively similar results in discriminating CVD to the other three IgM OSEs. Immunization of mice with MDA mimotope P1 has been shown to induce the generation of antibodies to MDA-LDL that strongly immunostained human atherosclerotic lesions (24). Since OSE epitopes are chemically complex, to date there has not been standardization of such antigens across laboratories, which leads to difficulty comparing studies in the literature. Although formal studies were not conducted to ascertain the sensitivity and specificity of the MDA mimotope to the other titers in this study, this can be further validated as a method to standardize such measurements in the future. The P1 mimotope is also commercially available and inexpensive.

We did not measure antiphospholipid antibodies in this study, but it is possible some of these IgM OSE have effects on thrombosis. For example, it has been demonstrated that the risk of recurrent thrombosis was higher in patients with low natural IgM, but not IgG, antibody levels (57). Furthermore, a subset of circulating microvesicles, a class of extracellular vesicles that are increasingly recognized as mediators of coagulation and biomarkers of thrombotic risk, are characterized by the presence of OSE and bound by natural immunoglobulin M (IgM) antibodies targeting these structures. It was demonstrated that the extent of plasma coagulation was inversely associated with the levels of both free and microvesicle-bound endogenous IgM (58). Moreover, the oxidation epitope-specific natural IgM antibody LR04, which recognizes malondialdehyde adducts, similar to IgM OSE MDA-LDL and MDA P1 mimotope, reduced microvesicle-dependent plasmatic coagulation and whole blood clotting without affecting thrombocyte aggregation. Intravenous injection of LR04 protected mice from microvesicle-induced pulmonary thrombosis. Of note, LR04 completed the binding of coagulation factor X/Xa to MVs, providing a mechanistic explanation for its anticoagulatory effect.

The determinants of plasma IgM to OSE are not known, but genetic predisposition may be a factor to some extent. Natural IgMs with specificity for OSE appear to constitute 20%–30% of all IgM in the absence of acute infection, in both mice and humans (59), suggesting that IgMs to OSE are critical to the host for cellular/organ homeostasis and protection (6, 60). Mice subjected to splenectomy, which dramatically reduces the plasma levels of innate IgM, have an approximately sevenfold higher risk of atherosclerosis, which was shown to be due to the absence of B-1 cells, including B-1a, making antibodies to OSE (61). B-1b cells were also shown to be capable of generating atheroprotective IgM antibodies against OSE (60). The spleen is the predominant source of innate IgM in humans and a long-term follow-up of 740 American servicemen who suffered a traumatic splenectomy revealed a nearly two-fold increased risk of dying of ischemic heart disease, without an excess in thromboembolism (62). IgM specific for OSE are thought to reflect innate NAbs that develop during fetal development and are present at birth or shortly thereafter even in the absence of antigen exposure, and their antibody variable genes have high homology to germline sequences with few if any non-templated nucleotide additions (13). Our prior observation that IgM to OSE has a high heritability score (heritability index h^2^ = 0.69–0.80) (63) suggests that there is a genetic predisposition to generate higher titers of such IgM to bind and inactivate OSE (64).

A clinical implication of this study is the measurement of IgM OSE may be developed as risk biomarkers independent of traditional risk factors and biomarkers. There are also potential therapeutic implications in that such antibodies modified for human use may offer protection to patients at risk for CVD. For example, infusion of the human oxidation-specific antibody IK17 that blocks the uptake of OxLDL by macrophages, either as a Fab or single chain, significantly reduces the progression of experimental atherosclerosis (33). Similarly, infusion of E06 IgM reduces liver ischemia–reperfusion injury (42) and acute pain syndromes (40), and administration of the MDA-specific IgM LR04, which was used to generate the MDA-mimotope P1, protects mice from extracellular vesicle-induced thrombosis (58). Furthermore, mice overexpressing a single-chain fragment of the murine monoclonal IgM antibody E06 have reduced myocardial infarct size following ischemia–reperfusion injury (65), a marked reduction in the progression of atherosclerosis, lower aortic valve gradients (6), reduction in manifestations of non-alcoholic hepatosteatosis (10), and improved bone metabolism (34, 36).

Limitations of this study include the fact that this is a case–control study, and although baseline variables were matched, there may be potential confounding not captured in the database. It is also known that these OSE markers can act as acute phase reactants and rise by 6–24 h following ACS (17) or percutaneous coronary intervention (19, 66). In the current study, blood samples were collected on hospital admission, limiting this potential confounding variable. Finally, the population is specific to patients from South Asia and these findings need to be confirmed in other populations.

In conclusion, higher titers of 4 IgM OSE markers were independently associated with a lower risk of AMI and provided independent risk prediction and risk discrimination in addition to traditional risk factors.

## Data availability

Allowable data that support the findings of this study may be made available from the corresponding author upon request.

## Supplemental data

This article contains supplemental data.

## Conflict of interest

S. T and J. L. W. are co-inventors and receive royalties from patents owned by University of California San Diego (UCSD) and are co-founders and have an equity interest in Oxitope, Pharmaceuticals and Kleanthi Diagnostics. Although these relationships have been identified for conflict-of-interest management based on the overall scope of the project, the research findings included in this publication may not necessarily relate to the interests of the above companies. The terms of this arrangement have been reviewed and approved by the University of California, San Diego in accordance with its conflict-of-interest policies. S. T. has a dual appointment at UCSD and Ionis Pharmaceuticals. J. L. W. is a consultant to Ionis Pharmaceuticals. The other co-authors have nothing to disclose.
