# Supplementary material for: High immunoglobulin-M levels to oxidation-specific epitopes are associated with lower risk of acute myocardial infarction
Source: J Lipid Res. 2023 May 19;64(6):100391. doi: 10.1016/j.jlr.2023.100391 (PMC10275726; doi:10.1016/j.jlr.2023.100391)

**Total Immunoglobulin Levels and Odds Ratios for CAD in EPIC-Norfolk**

The baseline mean (SD) total IgM [112 (75) vs. 116 (68) mg/dL, p=0.75], IgG [883 (231) vs. 885 (234) mg/dL, p=0.94], and IgA [218 (97) vs. 220 (95) mg/dL, p=0.47] levels were not significantly different in cases versus controls.

Total IgM correlated modestly with IgM MDA-LDL (r=0.570, p<0.001) and apoB-IC (r=0.438, p<0.001), but not with IgG MDA-LDL (r=0.032, p=0.112) or IgG ApoB-IC (r=0.005, p=0.795). Total IgG correlated weakly with IgG MDA-LDL (r=0.163, p<0.001) and IgG apoB-IC (r=0.307, p<0.001) and IgM ApoB-IC (r=0.079, p<0.001), but not with IgM MDA-LDL (r=0.020, p=0.315). Total IgA had weak to no correlations with all 4 IgM OSE: IgG MDA-LDL (r=0.046, p=0.022), IgG apoB-IC (r=0.090 p<0.001), IgM MDA-LDL (r=-0.066, p=0.112) and IgM ApoB-IC (r=-0.012, p=0.534).

Total IgM correlated with IgG (r=0.174, p<0.001) and total IgA (r=0.084, p<0.001) and total IgA correlated with total IgA (r=0.307, p<0.001).

In age, sex and smoking adjusted analyses, there was no significant association of total IgM, IgG and IgA and risk of CAD (**Supplemental** **Table**).

**Supplemental Table.** Predictive value of CAD according to quintiles for total IgM, IgG and IgA added to

conventional risk factors in the EPIC-Norfolk Study.

| Variables | Q1 | Q2 | Q3 | Q4 | Q5 | P-value |
| --- | --- | --- | --- | --- | --- | --- |
|  | Age, sex and smoking adjusted | | | | | |
| Total IgM | 1.00 | 1.04 (0.78-1.39) | 1.13 (0.85-1.52) | 1.14 (0.85-1.54) | 1.21 (0.90-1.63) | 0.73 |
| Total IgG | 1.00 | 1.15 (0.86-1.54) | 0.92 (0.68-1.23) | 1.08 (0.81-1.44) | 0.97 (0.73-1.30) | 0.57 |
| Total IgA | 1.00 | 0.83 (0.62-1.11) | 0.79 (0.60-1.05) | 0.90 (0.67-1.20) | 0.97 (0.74-1.29) | 0.42 |

**Supplemental Figure 1. Distribution of IgM oxidation-specific epitope titers over 24 hours since onset of chest pain.**

The panels depict the association of changes in log transformed IgM titers to autoantibodies against PC-BSA and MDA-LDL and apoB-immune complexes plotted against time of blood sampling over 24 hours from the onset of chest pain.


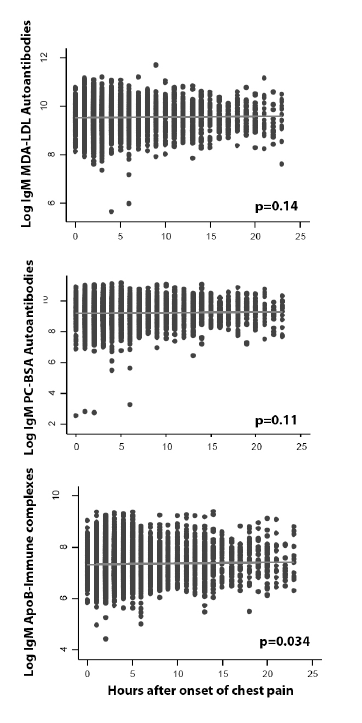


**Supplementary Figure 2. Decile analysis of IgM OSE markers**

The shape of the association of IgM OSe to myocardial infarction was analyzed in more detail by deciles. The data reveals that the odds ratios for myocardial infarction decrease continuously over most deciles, including all deciles in IgM PC-BSA. There is does not appear to be evidence for a threshold at a specific level.


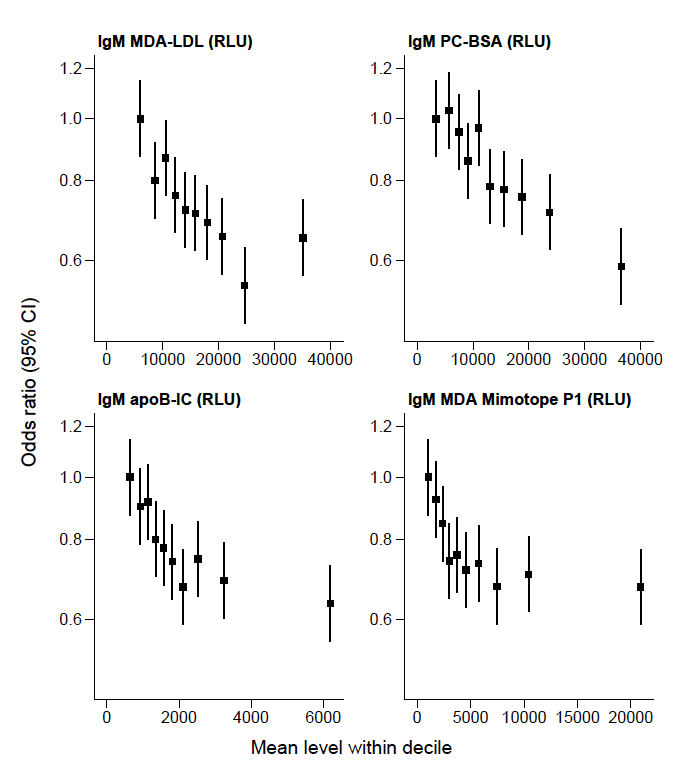

Supplement: Supplemental Table S1 and Figures S1 and S2 [file mmc1.docx]
